# Spirulina as Animal Feed: Opportunities and Challenges

**DOI:** 10.3390/foods11070965

**Published:** 2022-03-26

**Authors:** Brianne A. Altmann, Simon Rosenau

**Affiliations:** Department of Animal Sciences, University of Goettingen, 37077 Goettingen, Germany; simon.rosenau@agr.uni-goettingen.de

**Keywords:** microalgae, alternative protein source, animal nutrition, product quality, meat science, soybean meal replacement, fishmeal replacement

## Abstract

Increasing demand for protein, especially animal-based proteins and the large amounts of protein feed inputs required for production, has largely driven the research on spirulina as an animal feed. This short communication summarizes the results from two larger research projects investigating spirulina as an animal feed. Overall, spirulina appears to be a prospective protein source in poultry and pork production, as well as aquaculture. However, spirulina as a feed can have implications for system productivity and end product quality, depending on animal production system. Neither swine productivity nor product quality was negatively affected with spirulina as a feed, which is likely due to the low amounts of protein required in swine finishing diets. Spirulina as a feed does negatively affect poultry and fish productivity as well as alter product quality, primarily raw meat color. Therefore, future research focused on sustainability analysis and product processing and acceptance should investigate the trade-offs of incorporating spirulina into poultry and fish diets.

## 1. Introduction

Meat production requires large amounts of inputs and is therefore in many countries and cultures considered a high value food product. Yet, due to increasing wages and a global population, global demand for animal-based proteins continues to grow [1]. Spirulina (*Arthrospira platensis*) has moved into the spotlight as a sustainable source of protein for direct consumption [2] as well as animal feed [3]. The attention on spirulina stems from its high crude protein content (>60% in dry matter content) and as a source of essential amino [4] and fatty acids [5]. Furthermore, as a cyanobacteria, spirulina presents an interesting opportunity to utilize resources otherwise not included in the food production system. Spirulina can be cultivated free of arable land, in bioreactors or open ponds [6]. Utilizing alternative resources will be important in ensuring a sustainable development of food production systems to meet growing global protein demands [7].

As a part of multiple larger research projects, the Department of Animal Sciences at the University of Goettingen has thoroughly investigated spirulina as an animal feed ingredient. The project “Sustainability Transitions in Food Production” investigated the opportunities and barriers of spirulina transitioning into European animal production. In the project “Sustainable Trout Aquaculture Intensification (SusTAIn)” alternative proteins such as spirulina were investigated for their possible effects on growth parameters and product quality traits in trout and other commercially important fish species. This communication summarizes the findings of these projects.

## 2. Spirulina in Poultry Feed

Through a series of feed trial, Neumann et al. [8] investigated the graded inclusion of spirulina into broiler diets. The authors were successfully able to fully substitute soybean meal for spirulina; experimental diets were supplemented and balanced according to animal amino acid requirements [8]. Although a successful substation and incorporation of spirulina without adverse effects to animal growth was shown to be possible within numerous studies [8,9,10,11], lower live weights and decreased feed intake were observed when animal diets including spirulina were not balanced according to amino acid requirements [9].

Thereafter, the effect of spirulina as a feed on resulting meat quality was investigated. First, Altmann et al. [12,13] monitored meat quality using numerous physico-chemical parameters as well as sensory analysis. Perceivable differences in meat quality were identified in broiler chickens fed spirulina. In accordance with previous research [14,15], Altmann et al. [12,13] observed a more intense color of meat produced with spirulina as a feed; both breast and thigh meat exhibits higher red (a*) and yellow (b*) hues as recorded with the CIELAB color system. In other words, poultry meat turns an intense orange color when spirulina is included at high rates as a feed ingredient [16]. Based on this observation, research into implications regarding marketability was conducted. A study on consumer preferences for poultry meat appearance found that consumers will reject (not purchase) poultry meat produced with spirulina unless information explaining the unfamiliar color is provided [16].

Other subtler changes in meat quality included: increased umami and therefore chicken flavor [13] and decreased off-flavor [12,13] in breast meat produced with spirulina as a feed. Although spirulina is often cited as an antioxidant [5], meat samples produced with spirulina as a feed exhibited higher rates of lipid oxidation compared to other (soybean meal or insect meal) treatment groups, especially when meat samples were packaged in highly oxygenated modified atmosphere packaging [13]. Evaluation of meat quality also included monitoring fatty acid composition of intramuscular fat. Despite its reputation as a good source polyunsaturated fatty acids (PUFA), particularly gamma-linolenic acid (GLA) [5], spirulina as a feed did not result in higher GLA nor omega-3 levels in intramuscular fat, when comparing with meat samples raised on soybean meal [13]. The reason for stagnant levels of GLA remains unknown; even as GLA made up approx. 1% of fatty identifiable fatty acids in spirulina-based diets [13]. PUFA levels did not increase compared to soybean meal because soybeans are also a good source of PUFA and levels were similar across experimental diets [13]. Future research should focus on better understanding the physiological uptake of spirulina-derived fatty acids.

Gkarane et al. also conducted in-depth analysis on the effect of spirulina as a feed on meat aroma [17] and flavor precursors [18]. Spirulina as a feed decreased levels of endogenous bioactive compounds (i.e., anserine, creatine and carnosine); whereas it increased amounts of flavor-related compounds (i.e., inosine and insosine-5′-monophosphate) [18]. Furthermore, the aroma profile of spirulina fed chicken was found to be distinguishable from samples reared on other feeds; the profile was partially characterized by compounds associated with lipid oxidation [17].

These above-mentioned studies illustrate that spirulina can be successfully incorporated into poultry diets. However, due to amino acid requirements of poultry, spirulina is not sufficient in itself as a protein source; amino acid supplementation is required. This has implications for use in organic or low-input rearing systems. Furthermore, spirulina as a feed affects meat quality, in ways that are subjectively positive (e.g., increased flavor compounds and perceptible flavor and intensified color). Yet its effect as an antioxidant and its effect on improving fatty acid composition remain contested according to the results of this study.

## 3. Spirulina in Swine Feed

Based on the research carried out in the “Sustainability Transitions” project, spirulina appears to be a good candidate as a protein source in swine diets. Two studies investigated spirulina: one from an animal nutrition perspective and the other from the product quality perspective.

In their feeding trials with piglets and barrows, Neumann et al. [19] observed that soybean meal could be completely replaced by spirulina (with appropriate lysine supplementation) in swine diets without compromising overall protein quality. However, the authors also observed that supplementing histidine in high amounts when in combination with lysine, methionine and threonine improves protein quality of swine diets containing spirulina [19]. In additional trials, spirulina as a feed did result in slightly lower carcass weights, however, results remained insignificant compared to the soybean meal-fed group [20].

Furthermore, Altmann et al. [20] investigated the resulting product quality of barrows reared on diets containing spirulina as a protein source. In their study, finishing diets completely substituted soybean meal for spirulina; spirulina composed 9.5% of the ration [20]. Physico-chemical parameters, such as meat pH, water holding capacity, proximate composition, meat and fat color, lipid oxidation in meat and fatty acid composition of backfat were analyzed. Overall, spirulina at a rate of 9.5% of the finishing ration did not affect meat and fat quality compared to the control diet containing soybean meal [20]. One exception is that the backfat of spirulina fed barrows had a marginally lower proportion of monounsaturated fatty acids (MUFA) and increased PUFA compared to the soybean meal-fed control [20]. Unlike in the poultry experiment [13], a higher proportion of GLA was also found in the spirulina-fed samples [20]. However, the authors caution that these results should not be taken out of context; soybean oil was added in greater amounts to the experimental diets containing spirulina, which also likely confounded the effects of spirulina as a feed ingredient [20].

Sensory analysis was also conducted and showed that meat reared with a diet containing spirulina increased the overall odor of loin meat; spirulina as a feed was also associated with an increased astringent aftertaste in pork loin [20]. As results were marginal, the authors came to the conclusion that spirulina does not lead to drawbacks in product quality when included in swine feed [20].

Although only two pieces of literature were published, both include multiple replicates and different trials, illustrating the robustness of results. Spirulina can be included in swine feed at high rates, with appropriate amino acid supplementation, without disadvantaging animal nutrition or product quality.

## 4. Spirulina in Fish Feed

The use of spirulina has already been tested for various fish species, but high substitution levels often lead to a reduction in growth performance and increase in feed conversion ratio in carnivorous fish [21]. The objective of the SusTAIn project was to evaluate a whole fishmeal substitution with spirulina in terms of animal growth and product quality. Therefore, Dietz et al. [22] developed two isoenergetic and amino acid balanced diets, based on the recommendation from the National Research Council [23] for trout. The control diet contained fishmeal, but in the experimental diet fishmeal was completely exchanged by spirulina. The authors tested the diets on different rainbow trout (*Oncorhynchus mykiss*) genetic lines but did not find significant interaction in growth parameters and feed conversion between breed and diet.

In feeding trials with rainbow trout, brook trout (*Salvelinus fontinalis*), brown trout (*Salmo trutta fario*) and African catfish (*Clarias gariepinus*), Rosenau et al. [24,25] investigated the acceptance and performance of experimental diet containing spirulina. Overall, acceptance for the diets was high across all species, with the exception of brown trout. The authors hypothesized that this species may have rejected the spirulina diet due to an aversion to unfamiliar flavor. Overall, the digestibility for both diets was high, but the feed conversion ratio was increased for spirulina-fed rainbow trout and brook trout and resulted in significantly lower growth rates in all species. A subsequent investigation of the intestinal microbiome in African catfish, using 16S rRNA sequencing, found only slight changes for some bacterial genus, but the overall microbial community structure was not affected by spirulina-diet [26].

Rosenau et al. [24,25] also investigated the spirulina-induced changes in product quality. The authors observed higher red (a*) and yellow (b*) coloration for skin and also for raw fillet, leading to a more yellow-orange coloration in spirulina treatment groups. This change in color could have a major effect on consumer acceptance. Preliminary results of an online consumer survey suggest that fish consumers are not put-off by the unfamiliar color. In fact, the orange-yellow coloration may be preferred in trout fillet. These results contrast with results pertaining to poultry consumers [16] and are currently being prepared for publication.

Another important criterion for product quality is the fatty acid composition, which is strongly influenced by diet. While in African catfish, the saturated fatty acids (SFA), MUFA and PUFA showed no significant differences [25], in salmonid fish, a significant reduction in PUFA was observed [24]. Among the PUFA, important long-chain fatty acids such as eicosapentaenoic acid (EPA) and docosahexaenoic (DHA) were reduced in spirulina treatment groups [24].

These results show that advantages pertaining to sustainability and even consumer preferences can be linked to spirulina substitution of fishmeal. However, animal growth and product quality, especially food nutritional aspects, may come at a cost when fishmeal is completely replaced by spirulina. In addition, the viability of replacing fishmeal with spirulina in fish production and its implications are highly dependent on each species of fish and its trophic level. Spirulina as a fish feed impacts fish production to a lesser extent in omnivorous rather than carnivorous fish species.

## 5. Opportunities and Challenges

Using spirulina as an animal feed enables the nutritional quality of food to be improved so as to meet human nutritional requirements. Although spirulina can be processed and consumed directly [27,28], it is often rejected in Western cultures due to unfamiliarity and organoleptic characteristics, i.e., flavor [27,29]. In addition, spirulina does not meet all essential requirements for human development; although it contains small amounts of vitamin B_12_, primarily pseudovitamin B_12_ is found in spirulina [30]. Including spirulina as a protein source in animal feed culminates in a nutritional end product; meat contains numerous essential fatty and amino acids, as well as vitamins, including vitamin B_12_ [31], important for human growth and development. Overall, both meat reared using spirulina as a feed and direct consumption of spirulina should be seen as part of a well-balanced diet.

However, before spirulina can become a mainstream animal feed ingredient, a few challenges need to be overcome. Firstly, spirulina remains very expensive compared to other protein feedstuffs, such as soybean meal [6,21]. Although, due to the exorbitant cost of fishmeal compared to other protein sources, with improvements in production efficiency and utilizing waste streams as production media, spirulina could quickly become competitive on the market for fish feed [21]. Secondly, the cultivation of spirulina has remained, with few exceptions, small-scale and primarily for the nutritional supplement sector [6]. Thirdly, although spirulina is often heralded as a sustainable source of protein, its environmental footprint is extremely variable based on the production system and regional climate and comparable protein source [32]. For example, spirulina is not more sustainable to produce than soybean meal [32]; however microalgae may be more sustainable than fishmeal, especially incorporated into salmonid diets [33]. Nonetheless, improvements are needed to make production more sustainable. To this end, research continues to improve sustainability by including biogas effluent [34] or wastewater [35] as nutrient sources. As spirulina requires warm temperature (35–37 °C) for cultivation [36], integrating waste heat sources, such as is produced during biogas production, also has the potential to optimize cultivation facilities within Europe [37]. Upscaling and optimizing production will play a big part in overcoming the challenges presented here. Finally, although spirulina has a high proportion of crude protein, improvements to protein quality could be possible through breeding and nutrition/production research, as has been the case for other feedstuffs, such as soybean and faba bean [38].

There are two main aspects to consider when evaluating the advantages of spirulina as an animal feed: first, production efficiency (animal growth) and meat quality; secondly, is its ability to substitute less sustainable protein sources, i.e., fishmeal. As illustrated in this article, spirulina can be incorporated into poultry and swine diets without largely forfeiting animal productivity and product quality. The same holds mostly true for omnivorous fish species, such as the African catfish. Regarding its sustainability potential, once optimized, spirulina could be produced with high production capacities, small space requirement and low energy and water consumption [39]. This would grant it a leg up on replacing other protein sources dependent upon arable land and fishmeal with its problems of by-catch and fossil fuel powered ships. However, although spirulina may have the greatest sustainability potential through its incorporation into carnivorous fish aquaculture, it also negatively impacts animal growth and end product nutritional quality. Research needs to investigate these trade-offs further. In addition to these system-wide implications, research on product color and consumer preferences highlights spirulina as a good possible pigment medium. The application of pigments may be pursued to influence consumer liking and acceptance, as well as customize an intrinsic product attribute into a marker for extrinsic characteristics [40], such as production system or feed type.

## References

[1] OECD-FAO (2021). OECD-FAO Agricultural Outlook 2021–2030.

[2] Wang Y., Tibbetts S.M., McGinn P.J. (2021). Microalgae as sources of high-quality protein for human food and protein supplements. Foods.

[3] Martins C.F., Ribeiro D.M., Costa M., Coelho D., Alfaia C.M., Lordelo M., Almeida A.M., Freire J.P.B., Prates J.A.M. (2021). Using microalgae as a sustainable feed resource to enhance quality and nutritional value of pork and poultry meat. Foods.

[4] Mišurcová L., Buňka F., Vávra Ambrožová J., Machů L., Samek D., Kráčmar S. (2014). Amino acid composition of algal products and its contribution to RDI. Food Chem..

[5] Gutiérrez-Salmeán G., Fabila-Castillo L., Chamorro-Cevallos G. (2015). Nutritional and toxicological aspects of spirulina (Arthrospira). Nutr. Hosp..

[6] Chen J., Wang Y., Benemann J.R., Zhang X., Hu H., Qin S. (2016). Microalgal industry in China: Challenges and prospects. J. Appl. Phycol..

[7] Röös E., Bajželj B., Smith P., Patel M., Little D., Garnett T. (2017). Greedy or needy? Land use and climate impacts of food in 2050 under different livestock futures. Glob. Environ. Chang..

[8] Neumann C., Velten S., Liebert F. (2018). The graded inclusion of algae (*Spirulina platensis*) or insect (*Hermetia illucens*) meal as a soybean meal substitute in meat type chicken diets impacts on growth, nutrient deposition and dietary protein quality depending on the extent of amino acid Supple. Open J. Anim. Sci..

[9] Velten S., Neumann C., Bleyer M., Gruber-Dujardin E., Hanuszewska M., Przybylska-Gornowicz B., Liebert F. (2018). Effects of 50 percent substitution of soybean meal by alternative proteins from *Hermetia illucens* or *Spirulina platensis* in meat-type chicken diets with graded amino acid supply. Open J. Anim. Sci..

[10] Neumann C., Velten S., Liebert F. (2017). Improving the dietary protein quality by amino acid fortification with a high inclusion level of mi-cro algae (*Spirulina platensis*) or insect meal (*Hermetia illucens*) in meat type chicken diets. Open J. Anim. Sci..

[11] Velten S., Neumann C., Schäfer J., Liebert F. (2018). Effects of the Partial Replacement of Soybean Meal by Insect or Algae Meal in Chicken Diets with Graded Amino Acid Supply on Parameters of Gut Microbiology and Dietary Protein Quality. Open J. Anim. Sci..

[12] Altmann B.A., Neumann C., Velten S., Liebert F., Mörlein D. (2018). Meat quality derived from high inclusion of a micro-alga or insect meal as an alternative protein source in poultry diets: A pilot study. Foods.

[13] Altmann B.A., Wigger R., Ciulu M., Mörlein D. (2020). The effect of insect or microalga alternative protein feeds on broiler meat quality. J. Sci. Food Agric..

[14] Toyomizu M., Sato K., Taroda H., Kato T., Akiba Y. (2001). Effects of dietary Spirulina on meat colour in muscle of broiler chickens. Br. Poult. Sci..

[15] Venkataraman L.V., Somasekaran T., Becker E.W. (1994). Replacement value of blue-green alga (*Spirulina platensis*) for fishmeal and a vitamin-mineral premix for broiler chicks. Br. Poult. Sci..

[16] Altmann B.A., Anders S., Risius A., Mörlein D. (2022). Information effects on consumer preferences for alternative animal feedstuffs. Food Policy.

[17] Gkarane V., Ciulu M., Altmann B.A., Schmitt A.O., Mörlein D. (2020). The effect of algae or insect supplementation as alternative protein sources on the volatile profile of chicken meat. Foods.

[18] Gkarane V., Ciulu M., Altmann B.A., Mörlein D. (2020). Effect of alternative protein feeds on the content of selected endogenous bioactive and flavour-related compounds in chicken breast meat. Foods.

[19] Neumann C., Velten S., Liebert F. (2018). N balance studies emphasize the superior protein quality of pig diets at high inclusion level of algae meal (*Spirulina platensis*) or insect meal (*Hermetia illucens*) when adequate amino acid supplementation is ensured. Animals.

[20] Altmann B.A., Neumann C., Rothstein S., Liebert F., Mörlein D. (2019). Do dietary soy alternatives lead to pork quality improvements or drawbacks? A look into micro-alga and insect protein in swine diets. Meat Sci..

[21] Ragaza J.A., Hossain M.S., Meiler K.A., Velasquez S.F., Kumar V. (2020). A review on spirulina: Alternative media for cultivation and nutritive value as an aquafeed. Rev. Aquac..

[22] Dietz C., Spnder A., Liebert F. Does genetic background of Rainbow trout impact on growth and feed utilization following fishmeal substitution by partly defatted insect meal (*Hermetia illucens*) or microalgae powder (*Arthrospira platensis*)?. Proceedings of the 74rd Tagung der Gesellschaft für Ernährungsphysiologie.

[23] National Research Council (2011). Nutrient Requirements of Fish and Shrimp.

[24] Rosenau S., Ciulu M., Reimer C., Mott A.C., Tetens J., Mörlein D. (2022). Feeding green: Spirulina (*Arthrospira platensis*) induced changes in production performance and quality of three salmonid fish species. Aquac. Res..

[25] Rosenau S., Oertel E., Dietz C., Wessels S., Tetens J., Mörlein D., Ciulu M. (2021). Total replacement of fishmeal by spirulina (*Arthrospira platensis*) and its effect on growth performance and product quality of african catfish (*Clarias gariepinus*). Sustainability.

[26] Rosenau S., Oertel E., Mott A.C., Tetens J. (2021). The effect of a total fishmeal replacement by *Arthrospira platensis* on the microbiome of african catfish (*Clarias gariepinus*). Life.

[27] Grahl S., Strack M., Weinrich R., Mörlein D. (2018). Consumer-oriented product development: The conceptualization of novel food products based on spirulina (*Arthrospira platensis*) and resulting consumer expectations. J. Food Qual..

[28] Grahl S., Palanisamy M., Strack M., Meier-Dinkel L., Toepfl S., Mörlein D. (2018). Towards more sustainable meat alternatives: How technical parameters affect the sensory properties of extrusion products derived from soy and algae. J. Clean. Prod..

[29] Grahl S., Strack M., Mensching A., Mörlein D. (2020). Alternative protein sources in Western diets: Food product development and consumer acceptance of spirulina-filled pasta. Food Qual. Prefer..

[30] Watanabe F., Katsura H., Takenaka S., Fujita T., Abe K., Tamura Y., Nakatsuka T., Nakano Y. (1999). Pseudovitamin B12 is the predominant cobamide of an algal health food, spirulina tablets. J. Agric. Food Chem..

[31] Gille D., Schmid A. (2015). Vitamin B12 in meat and dairy products. Nutr. Rev..

[32] Smetana S., Sandmann M., Rohn S., Pleissner D., Heinz V. (2017). Autotrophic and heterotrophic microalgae and cyanobacteria cultivation for food and feed: Life cycle assessment. Bioresour. Technol..

[33] Draganovic V., Jørgensen S.E., Boom R., Jonkers J., Riesen G., Van Der Goot A.J. (2013). Sustainability assessment of salmonid feed using energy, classical exergy and eco-exergy analysis. Ecol. Indic..

[34] Hultberg M., Lind O., Birgersson G.G., Asp H.H. (2017). Use of the effluent from biogas production for cultivation of Spirulina. Bioprocess Biosyst. Eng..

[35] Olguín E.J., Galicia S., Mercado G., Pérez T. (2003). Annual productivity of Spirulina (Arthrospira) and nutrient removal in a pig wastewater recycling process under tropical conditions. J. Appl. Phycol..

[36] Habib M.A.B., Parvin M., Huntington T.C., Hasan M.R. (2008). A Review on Culture, Production and Use of Spirulina as Food for Humans and Feeds for Domestic Animals and Fish.

[37] Taelman S.E., De Meester S., Van Dijk W., da Silva V., Dewulf J. (2015). Environmental sustainability analysis of a protein-rich livestock feed ingredient in The Netherlands: Microalgae production versus soybean import. Resour. Conserv. Recycl..

[38] Stein H.H., Lagos L.V., Casas G.A. (2016). Nutritional value of feed ingredients of plant origin fed to pigs. Anim. Feed Sci. Technol..

[39] Singh R.N., Sharma S. (2012). Development of suitable photobioreactor for algae production—A review. Renew. Sustain. Energy Rev..

[40] Ardeshiri A., Rose J.M. (2018). How Australian consumers value intrinsic and extrinsic attributes of beef products. Food Qual. Prefer..

